# Towards dark current suppression in metallic photocathodes by selected-area oxidation

**DOI:** 10.1016/j.heliyon.2024.e31461

**Published:** 2024-05-22

**Authors:** C. Benjamin, S.D. Seddon, M. Walker, L.B. Jones, T.C.Q. Noakes, G.R. Bell

**Affiliations:** a Department of Physics, University of Warwick, Coventry, CV4 7AL, United Kingdom; b Institute of Applied Physics, Technische Universität Dresden, Dresden, 01069, Germany; c ASTeC, STFC Daresbuy Laboratory, Daresbury, WA4 4AD, United Kingdom; d Cockcroft Institute, STFC Daresbuy Laboratory, Daresbury, WA4 4AD, United Kingdom

**Keywords:** Dark current, Photocathode, Kelvin probe, Surface oxides

## Abstract

Oxide-free surfaces of polycrystalline Cu are prepared using acetic acid etching after chemical-mechanical polishing. UV ozone treatment is shown to increase the work function of the cleaned Cu by up to 0.5 eV. There is also a large reduction in quantum efficiency at 265 nm. Cu sheet can be easily masked from ozone exposure by Si or glass, meaning that selected-area oxi-dation is possible. Oxygen plasma treatment has a similar effect to the UV ozone but is more difficult to mask. There is no increase in surface roughness after oxidation, meaning that the larger work function could significantly re-duce dark current in accelerator photocathodes without affecting the desired photoemission region.

## Highlights

• UV ozone treatment increases the work function of clean Cu by up to 0.5 eV.
• A simple mask of silica glass or Si protects the Cu from ozone exposure.
• Prospects for dark current reduction in accelerators are discussed.
• An efficient polish-etch method for surface oxide-free Cu is given.

## 1 Introduction

**Fig. 1:**
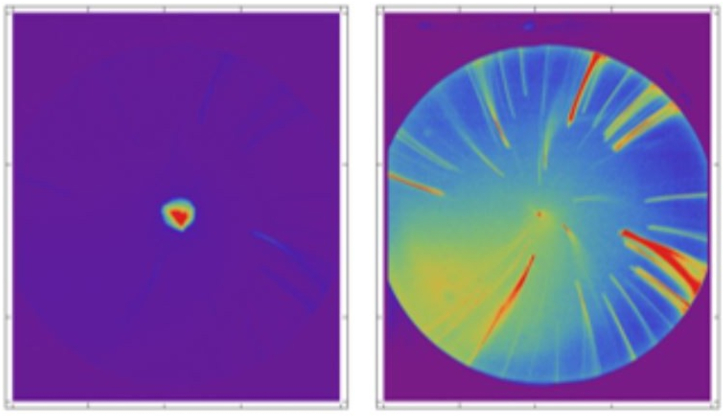
Typical currents observed on an yttrium aluminium garnet screen at the CLARA accelerator. Left: at 100 pC bunch charge. Right: without beam, but at 20 times higher camera sensitivity.

Modern accelerators typically use photoemission to generate electrons because it provides the flexibility to achieve the required time structure in the beam, in particular ultra-short pulses. However, in the high field envi-ronment of either a DC or RF photoinjector it is also possible to generate electrons via unwanted field emission, which is known as dark current. Field emission does not have the correct time structure and could produce a range of electron energies depending on the RF phase at which individual elec-trons are emitted. These electrons are undesirable for several reasons. They can provide an unwanted background signal or potentially hit other parts of the accelerator thereby generating X-rays and leading to electron-stimulated desorption of residual gases which can contaminate the vacuum system. The suppression of dark current is therefore an important consideration in pho-tocathode design. Fig. 1 illustrates dark current emitted from a standard copper photocathode puck used in the CLARA accelerator [1]. The red regions represent high current. Without beam (right panel), significant dark current emission associated with high-field regions in the electron gun can be observed. It is highly desirable to suppress such dark current without affecting the photoemission from the surface of the photocathode itself. One possibility is to increase the work function of all regions of the puck except the central section from which photoemission is desired. This would reduce field emission from these regions. In the case of GaAs photocathodes, selective area anodisation has been used to suppress electron emission from chosen regions of a target wafer in order to minimise ion backflow damage [2,3].

With an external electric field, the effective barrier for electron emission is reduced, and the barrier shape can be approximated reasonably simply [4]. The barrier correction factor *v*_*F*_ is given by

$$\upsilon_{F}=1-\frac{F}{F_{\Phi }}+\frac{F}{6F_{\Phi }}\mathrm{In}\frac{F}{F_{\Phi }}$$

where *F* is the external electric field. The external field which would drag the barrier height to zero (*v*_*F*_ = 0) depends on depends on work function Φ, and is denoted *F*_Φ_. The field emission current density *I* is then given by the usual tunnelling equation

$$I\propto \frac{F^{2}}{\Phi }\exp\left(-\upsilon_{F}b\varphi^{3/2}/F\right)$$

with *b* being the second Fowler-Nordheim constant.

The surface roughness of a photocathode may strongly affect its emission characteristics via local electric field enhancement or through reduction of the local work function [5]. Small-scale roughness and projections are thought to be particularly important for large-area photocathodes [[6], [7], [8], [9]]. In our calculations for this paper we assume a field enhancement factor at the low end of the 50–500 range typically cited [5].

In this paper we use selected-area oxidation to increase the work function of polycrystalline Cu samples. Both oxygen plasma and UV ozone treat-ments are used with simple masks in order to oxidise controlled areas of the Cu surfaces. We show that the work function of the oxidised regions can be increased by around 0.5 eV without affecting the masked areas. This is predicted to significantly reduce dark current under typical RF cavity conditions.

## 2 Methods

**Fig. 2:**
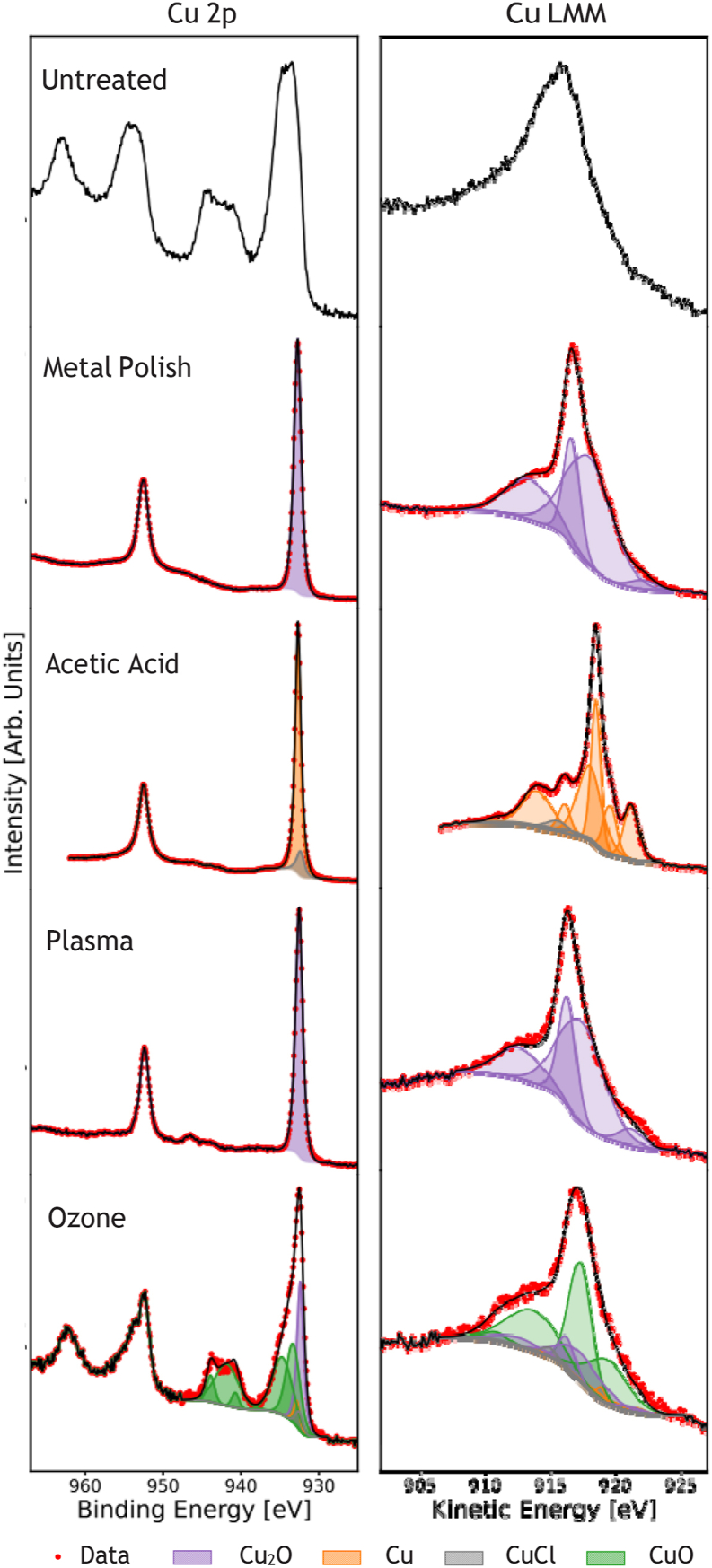
XPS spectra of the Cu 2p (left column) and the Cu LMM (right column) regions after sample preparation. Fitted components are shown as coloured regions (not included for untreated Cu).

Oxygen free copper foil (99.99 %, Advent Research Materials, UK) was cut into 10 mm square substrates. Samples were first chemically-mechanically polished with a household metal polish and soft cloth, which has two effects. Firstly, it removes any large-scale contamination and improves the surface finish compared to the as-supplied foils. Secondly, it removes the complex native oxides from the surface leaving a thin cuprous oxide layer. Excess metal polish and other residues were removed by acetone and isopropyl alchohol. The cuprous oxide layer was then removed by agitation in glacial acetic acid for 10 min, producing a pristine, oxide free copper surface [10]. The evolution of the oxide layer from as-supplied, through polished and acetic acid-cleaned was monitored using X-ray photoelectron spectroscopy (XPS), discussed below and shown in Fig. 2.

Once prepared, the samples were masked with either Si or glass pieces gently pressed on to the surfaces with clean stainless steel 5 g weights. Two methods of oxidation were investigated, namely oxygen plasma and ozone. Samples were exposed to an oxygen plasma generated using air as the feed gas to a standard plasma asher (Cambridge Fluid Systems Ltd., UK). Alternatively, ozone was generated from air using a high intensity mercury vapour lamp in a UV ozone cleaner (Ossila Ltd., UK). A range of exposure times was investigated for the two methods, with 10 min (plasma) and 30 min (ozone) chosen for the main Kelvin probe, quantum efficiency and XPS measurements. These times resulted in discoloration of the copper samples without significant changes of surface morphology.

The Kelvin probe system (KP Technology Ltd., UK) sits within an aluminium Faraday cage with volume 0.38 m^3^ and a dry nitrogen inlet facing the sample position. Measurements were made either under ambient air or under a continuous flow of N_2_ over the sample surface. The Faraday cage does not provide a fully inert atmosphere, but N_2_ flow serves to reduce the local humidity and re-oxidation rate of sample surfaces after wet chemical treatment and drying. The Kelvin probe uses a 2 mm diameter gold electrode which was calibrated periodically against a freshly cleaved highly oriented pyrolytic graphite sample [11], giving a probe work function in the range 4.60 ± 0.05 eV. Contact potential difference (CPD) is measured relative to this, with repeated measurements made every few seconds over a period of tens of minutes to several hours, in order to observe stabilization and/or evolution of the CPD. The random error in the stable CPD is significantly smaller than the variability in probe work function. We therefore assign an error of ±0.05 eV (around ± 1 %) to work functions. However, measurements made in quick succession are likely to have a smaller relative error. It was simple to place the probe on masked or unmasked regions of samples, using the discolouration as a guide.

The quantum efficiency (QE) measurement system [12] was situated in a high vacuum stainless steel chamber with a base pressure of 10^−7^ mbar. Photoemission is induced by a 265 nm UV LED (Roithner LaserTechnik GmbH), with a max radiated power of 0.5 mW and a spectral width of 13 nm.

The LED was situated within the chamber and was focused by a bifocal lens ensuring all light is incident to the sample. A collector cup surrounding the sample was negatively biased by 45 V with respect to the sample and the QE measured via the drain current from the sample using a Keithley model 6485 picoammeter. The high-field QE could differ significantly from that in the low-field measurement system, and so the apparatus was used to provide an indicative relative QE measurement between clean and oxidised Cu.

Light microscopy and atomic force microscopy (AFM) were used to study the surface morphology of the Cu samples. This is important for field emission through the enhancement of surface electric field at protrusions [13]. AFM images were analysed using the Gwyddion software package [14].

## 3 Results and discussion

XPS spectra were acquired at the Warwick Photoemission Facility after each treatment step. We show only the most relevant data concerning Cu oxidation: apart from Cu and O peaks only a small adventitious C 1s feature is observed. The evolution of Cu 2p and Cu LMM core region spectra is shown in Fig. 2. The Cu 2p region for untreated samples shows a com-plex spectrum with multiple chemical states, while the Cu LMM region is broadened and featureless. For treated samples, the Cu LMM line shape was fitted using the reference data from Biesinger et al. [15] while the Cu 2p_3/2_ peak was fitted with standard component lineshapes and Shirley background. Table 1 shows the line positions, modified Auger parameter (mAP), which is the sum of the two peak positions, and the amount-of-substance (AoS) quantification.

**Table 1:** Peak positions of the Cu 2p_3/2_ and Cu LMM features, and their resulting mod-ified Auger parameter (mAP) at each Cu treatment stage. The approximate amount-of- substance quantification is also given, including a rough estimate for the untreated Cu.

Treatment	Cu 2p_3/2_ (eV)	Cu LMM (eV)	mAP (eV)	AoS (%)
Untreated	933.4	916.3	1849.7	Cu_2_O ∼23
				CuO ∼44
				Cu(OH)_2_ ∼33
Metal polish	932.7	916.8	1849.5	Cu_2_O 100
Acetic acid	932.7	918.6	1851.3	Cu(0) ∼91
				CuCl ∼9
Ozone	932.5	917	1849.5	Cu_2_O ∼45
				CuO ∼55
Plasma	932.5	916.5	1849.0	Cu_2_O 100

After polishing and solvent rinsing, the Cu 2p_3/2_ peak can be fitted with a single component, and the Cu LMM peak with three components. Based on the LMM lineshape, the mAP of 1849.5 eV and the lack of satellite peaks in the Cu 2p region, this can be assigned to Cu_2_O. No metallic Cu or mixed oxidation state components are observed. Noting that 99.7 % of measured photoelectrons come from within three inelastic mean free path lengths of the surface [16], we deduce that the surface Cu_2_O layer thickness must be greater than 5 nm.

There is little change in the Cu 2p lineshape and position after acetic acid etching. A small CuCl component appears due to chloride impurities in the acetic acid, in agreement with the results of Chavez et al. [10]. The weighting of this component agrees with the observed Cl 2p peak intensity (not shown).

In contrast to the Cu 2p peak, the Cu LMM lineshape is drastically different, with the mAP shifting by nearly 2 eV compared to the Cu_2_O produced by the polishing step. This is consistent with complete removal of the oxide and the presence of metallic Cu.

Note that we have also confirmed that Ar ion sputtering of the acetic acid-etched Cu rapidly produces a pristine Cu surface free of oxides, adventitious carbon and CuCl. The chemical pre-treatment is highly effective in reducing the sputtering time required, making this an efficient recipe for producing atomically clean polycrystalline Cu surfaces.

In the case of oxygen plasma-treated copper, the lineshapes and Auger parameter indicate a pure Cu_2_O surface layer very similar to that found after polishing, and of sufficient thickness to mask metallic Cu contributions. The ozone-treated sample is very different. Multiple components and satellite peaks appear in the 2p region and the LMM lineshape shifts compared to other samples. This can be explained by a mix of Cu^1+^ and Cu^2+^ oxidation states. Quantitative fitting implies a surface region comprising approximately equal amounts of CuO and Cu_2_O. The total oxide thicknesses found here (> 5 nm) are in agreement with a recent study of oxygen plasma-cleaned Cu using XPS depth-profiling and medium energy ion scattering (MEIS), where oxide thicknesses of 10–20 nm were found [17]. However, different Cu chemical state behaviour was observed in that study, with Cu^2+^, ascribed to CuO_2_, resulting from oxygen plasma treatment. The Cu_2_O state found in the present work probably results from significant differences in the plasma treatment employed. In this work, lower powers and exposure times were used, but most importantly, pure oxygen was used as the process gas in Ref. [17], which likely resulted in a more strongly oxidising atmosphere capable of forming CuO_2_. The oxidative environment produced by the UV ozone cleaner is clearly different to that of either plasma treatment, resulting in mixed Cu^1+^ and Cu^2+^ states. Yang et al. studied the roles of ozone [18] and ClO_2_ [19] as oxidants for the efficient leaching of Cu_2_O (cuprite). They found that ozone exposure of natural cuprite led to a chemically shifted component 1.7 eV higher in binding energy, with the two components ascribed to Cu_2_O and CuO, plus a broader satellite peak. These new components were stronger for ClO_2_ treatment [19]. Our Cu-ozone XPS results are very similar. This can be ascribed to the initial surface layer of Cu_2_O being of sufficient thickness to behave like bulk cuprite to the surface-specific ozone reaction.

**Fig. 3:**
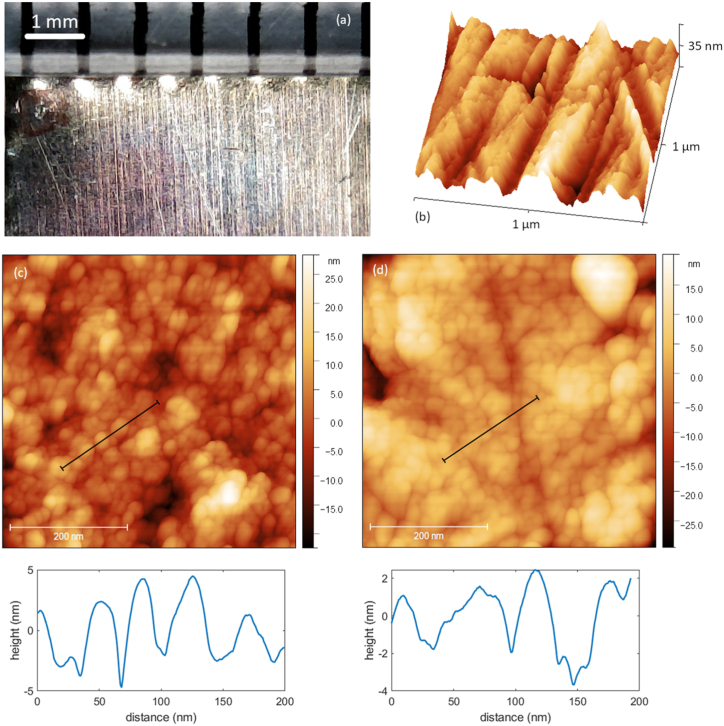
Microscopy of copper surface morphologies. (a) Light microscopy of large-scale morphology showing linear features from the manufacturing process along with scratches, as well as manual cutting features next to the mm scale ruler. (b) AFM topograph rendered in 3D of a typical region of a Cu sample after acetic acid etch. (c) AFM topograph with a line profile (black line on image, plotted below) of a region of a Cu sample after plasma oxidation. (d) The equivalent topograph and line profile for a Cu sample after ozone oxidation for 60 min.

Turning to the microscopy results, we find that the mm-scale morphology is dominated by grooves from the Cu sheet manufacturing process and scratches from manual handling. These features are highlighted in the light micrograph shown in Fig. 3(a). The bright dimples at approximately 0.8 mm intervals along the top of the sample are due to manual cutting with serrated cutters. This procedure can introduce slight non-planarity in samples. Grooves and scratches are reduced by polishing; we did not try to obtain very smooth surfaces through this manual process but instead achieved consistent large-scale morphology across the sets of samples. Smaller-scale features were examined by AFM. Topographs were obtained with image sizes from 2 μm to 100 nm, for two sets of Cu samples subject to different surface treatments after polishing and degreasing. These were (1) acetic acid etch, (2 and 3) acetic acid plus 30 or 60 min UV ozone exposure, and (4) acetic acid plus 10 min oxygen plasma treatment.

An example 3D rendering of the *μ*m-scale surface morphology for a sample treated by acetic acid etch is shown in Fig. 3(b), highlighting smaller scale linear features likely due to polishing. A height range of 30–40 nm across a 1 μm image is typical, though flatter areas could be readily found. In Fig. 3(c) and (d) are shown 500 nm AFM topographs of two Cu samples after plasma and ozone oxidation respectively. The rounded feature in the top right corner of (d) is probably surface contamination but otherwise the morphology is dominated by protrusions of a few nm height and widths of tens of nm, along with the linear features. Root-mean-square surface roughness values were typically 15–40 nm for 1 μm images and did not show any clear trend with surface preparation method. Overall, we found no evidence that the ozone and plasma surface treatments systematically affected the morphology across the length scales studied. This would very likely apply also to the surface morphology of a Cu photocathode puck, and therefore any changes to dark current arising from these treatments could be ascribed to alterations of the work function.

**Fig. 4:**
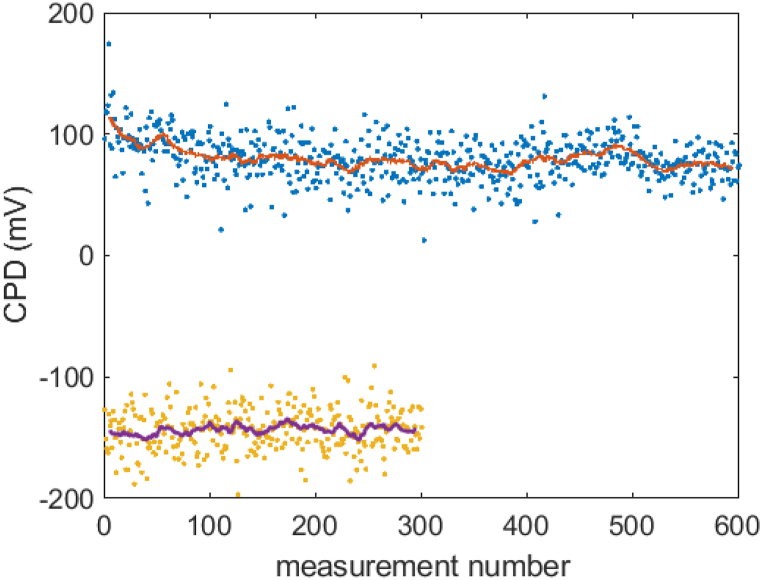
Typical traces for CPD measurements under ambient air. Points are individual CPD measurements while solid lines are 20-point moving averages. The samples have been oxidised by ozone treatment (30 min), and the top trace shows an unmasked region, while the lower trace shows a poorly masked (“leaky”) region.

The clean Cu work function was measured as 4.30±0.05 eV over several samples, i.e. the variation between samples was similar to the likely variation in probe absolute work function. Slightly different crystallographic texture from sample to sample could account for this, since the work function of Cu single crystals varies more widely with surface plane [20]. Variations in surface roughness could also play a role [21] although no significant changes were observed in our sample set. Example CPD time series are shown in Fig. 4 for an ozone-treated sample masked with Si. The CPD is steady at each of two positions measured, an unmasked region where full oxidation has occurred, and a poorly masked region where partial ozone exposure has occurred. The upper trace shows a positive CPD of 100 mV (work function 4.70 eV), while the lower trace shows a CPD of −140 mV (work function 4.46 eV). This shows that oxidation by UV ozone increases the Cu work function.

**Fig. 5:**
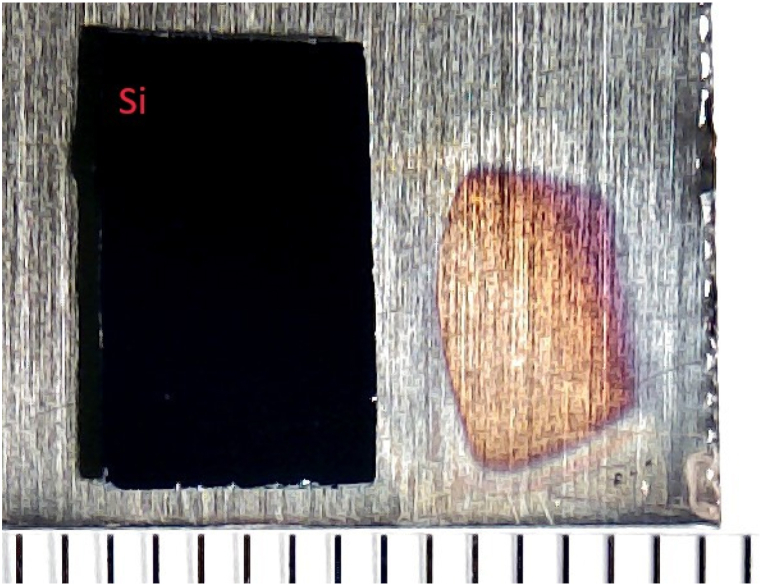
Photograph of a “leaky” mask adjacent to the area it originally covered during 15 min oxygen plasma treatment.

Table 2 gives the work functions for different treatments and masks using the exposure times specified in Sec. 2. Masked areas of the samples generally show very similar work function to clean Cu, i.e. the masks are effective in preventing oxidation. The table shows a value of 4.53 eV for plasma treatment with Si mask. Such “leaky” masks, where the masked region did not maintain its clean Cu work function of 4.3 eV, were sometimes observed on all combinations of samples, mask materials and oxidation methods, but most commonly for oxygen plasma treatment. We attribute them principally to non-planarity of the sample, allowing oxygen radicals to penetrate beneath the mask. The effect was seen far less often for UV ozone treatment. This is most likely due to the enhancement of chemical reactivity by direct UV illumination, which is easily blocked by even a loose mask (including the glass). The higher temperature of the plasma process might also allow the oxygen radicals to diffuse more efficiently. As well as changes of work function, leaky masks could be identified by the effect of oxidation treatment on the surface colouration of the samples, subtle in the case of UV ozone treatment but much more obvious for plasma treatment. Fig. 5 shows a photograph of a larger Si mask adjacent to the region it originally covered in oxygen plasma treatment (15 min). There is a clear gradation of colour with a smaller region of the masked area retaining the clean Cu hue. Despite such problems, even the very simple masking procedure adopted here could reliably and effectively shield a planar region of the sample from work function increase due to UV ozone treatment. It seems a practical proposition to fabricate an efficient, re-useable mask for a metal photocathode puck of well-defined dimensions.

**Table 2:** Work function values Φ for Cu samples after different treatments. The error bar is ±0.05 eV.

Treatment	Mask	Φ (eV)
Clean Cu	–	4.30
Ozone	None	4.80
Ozone	Si	4.29
Ozone	Glass	4.32
Plasma	None	4.64
Plasma	Si	4.53
Plasma	Glass	4.35

The relative QE was measured for four samples: polished and solvent-cleaned Cu, Cu subsequently acetic acid-etched, and Cu subsequently treated with ozone and plasma. The relative QE of the acetic acid-etched Cu was 2.3 times higher than that of the polished Cu. This is consistent with a reduced oxide thickness (some oxide could reform during transfer through air to the QE vacuum system) and work function for the acetic acid-treated surface. The typical absolute QE for chemically treated Cu surfaces is 7 10^−5^ [12]. No photocurrent could be measured for the two oxidised samples, i.e. the QE could not be distinguished from zero. This is consistent with a raising of the work function to 4.6–4.8 eV, close to or above the photon energy of 4.68 eV at 265 nm. It is plausible that a semiconducting surface oxide of thickness at least 5 nm reduces charge transport to the surface and hence suppresses QE beyond the effect due to work function increase alone. An additional potential barrier due to a semiconducting surface oxide would also reduce field-emission current, although this cannot be quantified by the present measurements.

**Fig. 6:**
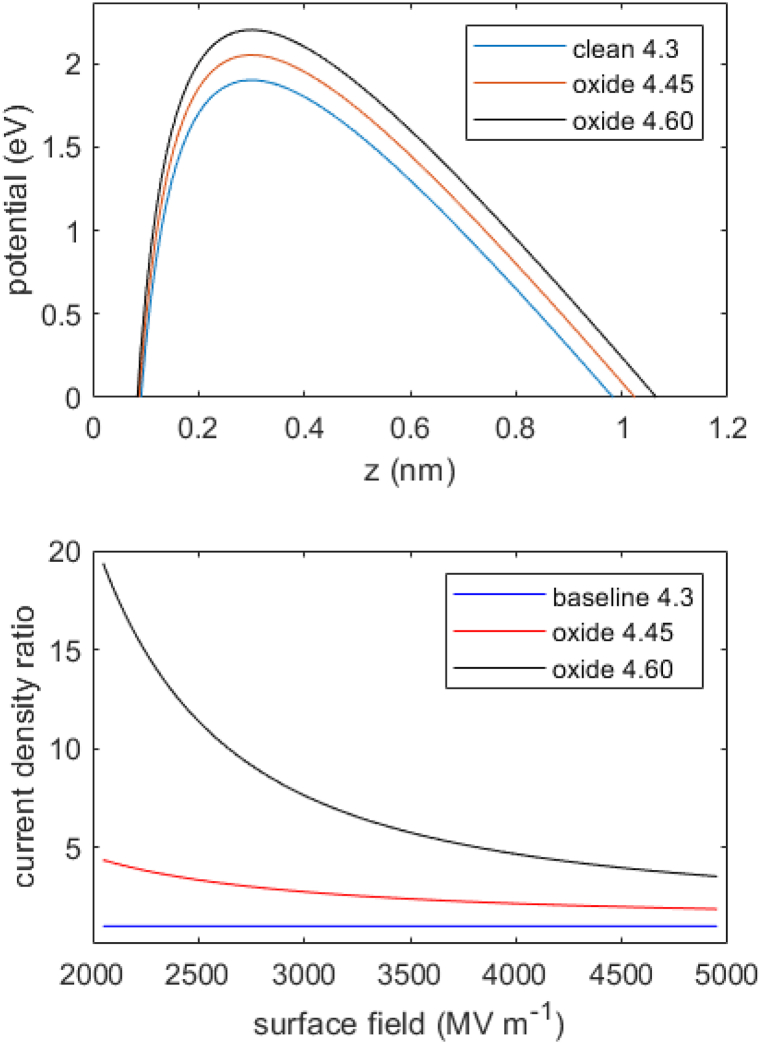
Upper panel shows the Schottky-Nordheim barrier shapes for work functions of 4.3, 4.45 and 4.6 eV, and surface field 4000 MV m^−1^. Lower panel shows the dark current density reduction ratio for the two oxide work functions, with the clean 4.3 eV surface as baseline.

It is possible to estimate the effect of work function on dark current using the field emission equations for a Schottky-Nordheim barrier. The barrier shape is shown in Fig. 6 at an assumed surface field of 4000 MV m^−1^ for three work functions, corresponding to two oxidised Cu surfaces plus the clean surface with work function 4.3 eV. The surface field reduces the barrier to 1.9 eV for clean Cu, but this is increased to 2.2 eV for the higher oxide work function. The increased barrier reduces the field emission current den-sity significantly. The decrease of current density for oxidised Cu is shown in the lower panel as a ratio, with the 4.3 eV clean work function as baseline. The reduction is by a factor of about 5 at 4000 MV m^−1^ for the higher work function and increases at lower surface fields. Note that for a typical S-band RF gun operating with a peak accelerating field 70–120 MV m^−1^, the value of 4000 MV m^−1^ corresponds to an enhancement of approximately 30–60. Surface features with such high aspect ratio are not observed on our samples (Fig. 3) and it may be the case that local work function lowering is a key mechanism [5]. This could be investigated directly using a spatially resolved probe of work function such as Kelvin probe force microscopy (KPFM). Indeed, studies of the effective doping of graphene on polycrystalline Cu suggest that subtle changes of surface morphology through annealing may affect the local work function [22]. Therefore, while the present work shows that overall surface roughness is minimally affected by the ozone and plasma treatments, and that average work function increases, further study of local work function would be useful.

## 4 Conclusion

This work suggests that deliberate oxidation could reduce dark current significantly over a useful range of surface fields. For both the plasma and ozone treatments used, the surface roughness is not significantly altered, meaning that geometric surface field enhancement should not occur. Both an increase of work function and a reduction of charge transport through the surface oxide layer should act to reduce dark current in treated areas. Simple physical masking of the laser target area of a photoemission puck to prevent oxidation seems eminently feasible. The simple masking techniques employed in this study sometimes resulted in “leakage” into masked areas, but accurately machined masks for photocathode pucks of precise dimensions should give improved shielding from oxidation. Our results suggest that UV ozone treatment is easier to mask successfully while providing equivalent or superior work function increase to oxygen plasma exposure. Further studies in realistic high field environments would advance understanding of the effects of selected-area oxidation. It would also be valuable to use KPFM to investigate small-scale work function variations on clean and oxidised Cu.

## Data availability

Data have not been deposited into a publicly available repository. Data will be made available on request.

## CRediT authorship contribution statement

**C. Benjamin:** Writing – review & editing, Validation, Investigation, Formal analysis. **S.D. Seddon:** Investigation. **M. Walker:** Writing – review & editing, Investigation, Funding acquisition, Formal analysis, Data curation. **L.B. Jones:** Writing – review & editing, Validation, Supervision. **T.C.Q. Noakes:** Writing – review & editing, Validation, Supervision, Project administration, Conceptualization. **G.R. Bell:** Writing – review & editing, Writing – original draft, Validation, Supervision, Project administration, Methodology, Investigation, Funding acquisition, Formal analysis, Conceptualization.

## Declaration of competing interest

The authors declare the following financial interests/personal relationships which may be considered as potential competing interests: Marc Walker reports financial support was provided by 10.13039/501100000266Engineering and Physical Sciences Research Council. If there are other authors, they declare that they have no known competing financial interests or personal relationships that could have appeared to influence the work reported in this paper.
